# A randomized, placebo-controlled trial investigating the acute and chronic benefits of American Ginseng (Cereboost®) on mood and cognition in healthy young adults, including in vitro investigation of gut microbiota changes as a possible mechanism of action

**DOI:** 10.1007/s00394-021-02654-5

**Published:** 2021-08-15

**Authors:** Lynne Bell, Adrian Whyte, Cindy Duysburgh, Massimo Marzorati, Pieter Van den Abbeele, Romain Le Cozannet, Pascale Fança-Berthon, Emilie Fromentin, Claire Williams

**Affiliations:** 1 School of Psychology & Clinical Language Sciences, University of Reading, Earley Gate, Whiteknights Road, Reading, RG6 6ES UK; 2 ProDigest BV, Ghent, Belgium; 3 Naturex SA, Avignon, France

**Keywords:** American ginseng, Cognition, Mood, Gut microbiome, SCFA

## Abstract

**Purpose:**

Cereboost®, an American ginseng extract, has shown improved short-term memory and attention/alertness in healthy young and middle-aged individuals, potentially via modulation of the gut microbiome and upregulation of neurotransmitters such as acetylcholine. Here, we explored the effects of Cereboost® on cognition and mood in the first 6 h post intervention (acute), after 2 weeks daily supplementation (chronic), and whether 2 weeks daily supplementation altered the response to a single acute dose (acute-on-chronic). A concurrent in vitro study evaluated effects of repeated Cereboost® administration on human gut microbiota.

**Methods:**

Cognitive effects of Cereboost® were assessed using a double-blind, randomized, placebo-controlled clinical trial, with 61 healthy young adults. Modulation of the gut microbiome was concurrently modelled using the Simulator of the Human Microbial Ecosystem (SHIME®), using a young adult donor.

**Results:**

Consistent with previous findings, Cereboost® improved working memory and attention during the immediate postprandial period; effects that were amplified following two weeks’ treatment (acute-on-chronic) compared to acute testing alone. Chronic supplementation improved cognition on an acetylcholine-sensitive attention task and improved mental fatigue and self-assurance aspects of mood. The parallel in vitro study revealed significantly increased acetate, propionate, and butyrate levels in simulated proximal and distal colon regions, linked with observed increases in *Akkermansia muciniphila* and *Lactobacillus*.

**Conclusion:**

This study confirmed the promising effects of Cereboost® on cognitive function and mood, while suggesting a possible link to alterations of the gut microbiome and modulation of acetylcholine. Further studies will be required to unravel the underlying mechanisms that are involved.

**Registration:**

The study was pre-registered at ClinicalTrials.gov on 6th July 2018 (Identifier: NCT03579095).

**Supplementary Information:**

The online version contains supplementary material available at 10.1007/s00394-021-02654-5.

## Introduction

Ginseng is a globally popular herbal root extract obtained from plants of the Panax family [1]. It is widely regarded as a panacea in traditional medicine and has been used for centuries to treat mental and physical ailments, and promote longevity [2]. Ginseng contains a number of bioactives including flavonoids and other phenolic compounds, essential oils, and vitamins [3]. However, the main bioactive ingredients in ginseng are purported to be ginsenosides including Rb1, Re, Rd, and F11. There are multiple species of ginseng which can be distinguished by their profiles of these ginsenoside subtypes [4]. American ginseng (*Panax quinquefolius*) provides a particularly rich source of these ginsenoside types when compared to other ginseng varieties [5].

Emerging research suggests that supplementation with *P. quinquefolius* may elicit cognitive enhancement effects. Studies have typically investigated the benefits of Cereboost®, a standardised extract of American ginseng, although only acute benefits have been investigated to date. For example, following acute supplementation with 100 mg, 200 mg and 400 mg Cereboost®, improvements on an immediate word recall task, a visuospatial working memory task, a choice reaction time task, a composite working memory factor, and a measure of subjective mood (i.e. calmness) were observed in healthy young adults (aged 18–40 years) during the six hours after consumption [6]. Adding to these findings, a study of middle-aged adults (aged 40–60 years) observed similar acute benefits of 200 mg Cereboost® on a composite working memory factor after three hours [7], although no changes in mood were observed in this older age group.

There is currently limited in vitro and in vivo research investigating possible mechanisms of action for observed improvements to mood and cognition following treatment with *P. quinquefolius*. Two mechanisms have been proposed to date; blood glucose regulatory effects [8–11], and effects on acetylcholine-related pathways of neurotransmission [12, 13]. The latter mechanism is of particular interest as Rb1, an abundant ginsenoside in *P.quinquefolius,* has been associated with upregulation of cholinergic pathways [14, 15]. Such cholinergic systems are known to be important in modulating cognitive functions [16], including learning and memory [4], and attention [17].

The timing and strength of cognitive benefits following treatment with ginseng are likely to be strongly dependent on the metabolism, and thus subsequent bioavailability, of the ginsenosides present. Ginsenosides, such as Rb1, are known to be absorbed from the upper gastrointestinal tract [18]. A study investigating the oral administration of American ginseng in healthy adults (age not specified) found Rb1 to be present in plasma samples throughout a 2–12-h period after administration [19]. Cognitive enhancement associated with American ginseng supplementation may also arise from compound K, an active metabolite of ginsenoside Rb1 [20], which is detectable in human plasma following oral consumption [21]. Rb1 is converted into compound K by intestinal bacteria through a process of deglycosylation and fatty acid esterification [22]. However, this process is dependent on an individual’s gut microbiota profile, which in turn may be determined by diet. For example, greater ratios of compound K to Rb1 have been observed in plasma, urine and faeces following consumption of American ginseng when regularly consuming a western diet rather than an Asian diet [23]. The regular consumption of ginseng is also likely to beneficially alter the gut microbiome [24, 25] and emerging evidence suggests that the microbiome is involved in brain development and cognitive function via the gut-brain axis. Mechanisms include production of neurotransmitters and short-chain fatty acid (SCFA) metabolites, that are implicated in brain function [26]. Positive changes to the microbiome may therefore also benefit cognitive function. However, this is yet to be investigated with respect to ginseng supplementation in a young-adult population.

The cognitive and mood benefits of *P. quinquefolius* remain under investigation, with exploration of repeated daily (chronic) supplementation seemingly a significant omission in the current datasets. Therefore, we aimed to investigate the acute, chronic, and acute-on-chronic benefits of 200 mg Cereboost® in healthy young adults (aged 18–40 years). This population has previously demonstrated sensitivity-to-acute supplementation with Cereboost®, during a six-hour period after consumption [6]. In the current study, testing of mood and cognitive function was therefore performed in the immediate postprandial period at 2 h, 4 h and 6 h following Cereboost® or placebo. These acute and acute-on-chronic test visits took place before and after daily supplementation for a pilot investigatory period of 14 days, respectively (Experiment 1). It was hypothesized that daily supplementation with Cereboost® would improve cognitive function and mood. It was also speculated that previously observed acute benefits to mood and cognition [6, 7] might be enhanced following the 14-day period of daily supplementation, as any beneficial changes in gut microbiota might lead to improved metabolism and subsequent bioavailability of the bioactive compounds such as ginsenosides present in Cereboost®. Although 14 days appears a relatively short intervention duration, changes to gut microbiota are known to occur relatively quickly, in only hours, or days following dietary changes [27]. To investigate whether Cereboost® might indeed impact gut microbiota during such a short timeframe, a concurrent in vitro study was performed (Experiment 2). The SHIME® technology platform was used to model changes in the human microbiome, using a faecal sample obtained from a healthy young adult donor, and following the same daily dosing with 200 mg Cereboost® for a similar intervention duration with weekly microbial sampling up to 21 days. It was hypothesized that the composition of the gut microbiota would be beneficially affected, resulting in a greater abundance of SCFA microbial metabolites within the simulated colon.

## Experiment 1

### Methods

#### Participants

Sample size was determined by power analysis assuming a moderate effect size (d = 0.65), obtained from a meta-analysis of *P.ginseng* effects on cognitive function [28]. A minimum of 60 participants were required to achieve a statistical power of 80% when comparing treatment and placebo group scores. An initial screening target of 80 allowed for 15% ineligibility or dropout. Following screening, 63 healthy participants, aged 18–40 years, 15 males, were recruited from students at University of Reading. Full demographic information is reported in Table 1. Exclusion criteria included food allergies, diabetes, psychiatric disorders, gastrointestinal disorders, and those taking any medication other than oral contraceptives. Participants were required to have a healthy BMI and to be non-smokers and non-vegetarians (due to the presence of gelatine in the intervention capsules). Participants were not permitted to take any additional supplements for the duration of the study, commencing at screening. Health criteria were determined via self-report questionnaire except for BMI which was measured by a researcher. Participants were requested to notify the researcher of any changes to their health or medication status over the course of the study.

**Table 1:** Participant demographic information

	Cereboost® group	Placebo group	*p* value
	(*n* = 30)	(*n* = 31)	
Age (years)	20.6 ± 2.4	20.5 ± 2.7	ns
Gender (F:M)	23:7	23:8	ns
Body weight (kg)	62.7 ± 9.4	63.7 ± 12.5	ns
Height (cm)	170.3 ± 8.4	166.5 ± 9.4	ns
BMI (kg/m^2^)	21.6 ± 2.3	22.9 ± 3.6	ns
Habitual fruit and veg consumption (portions/day)	5.9 ± 2.2	5.3 ± 2.8	ns
Habitual calorie intake (kCal/day)	1524 ± 424	1520 ± 629	ns
Habitual NSP fibre intake (g/day)	13.6 ± 2.5	14.5 ± 7.4	ns

Data presented as mean ± SD or frequency ratio, *p *values calculated using independent samples * t*-test or Chi-square statistic, ns indicates non-significance (*p* > 0.05)

### Design

**Fig. 1:**
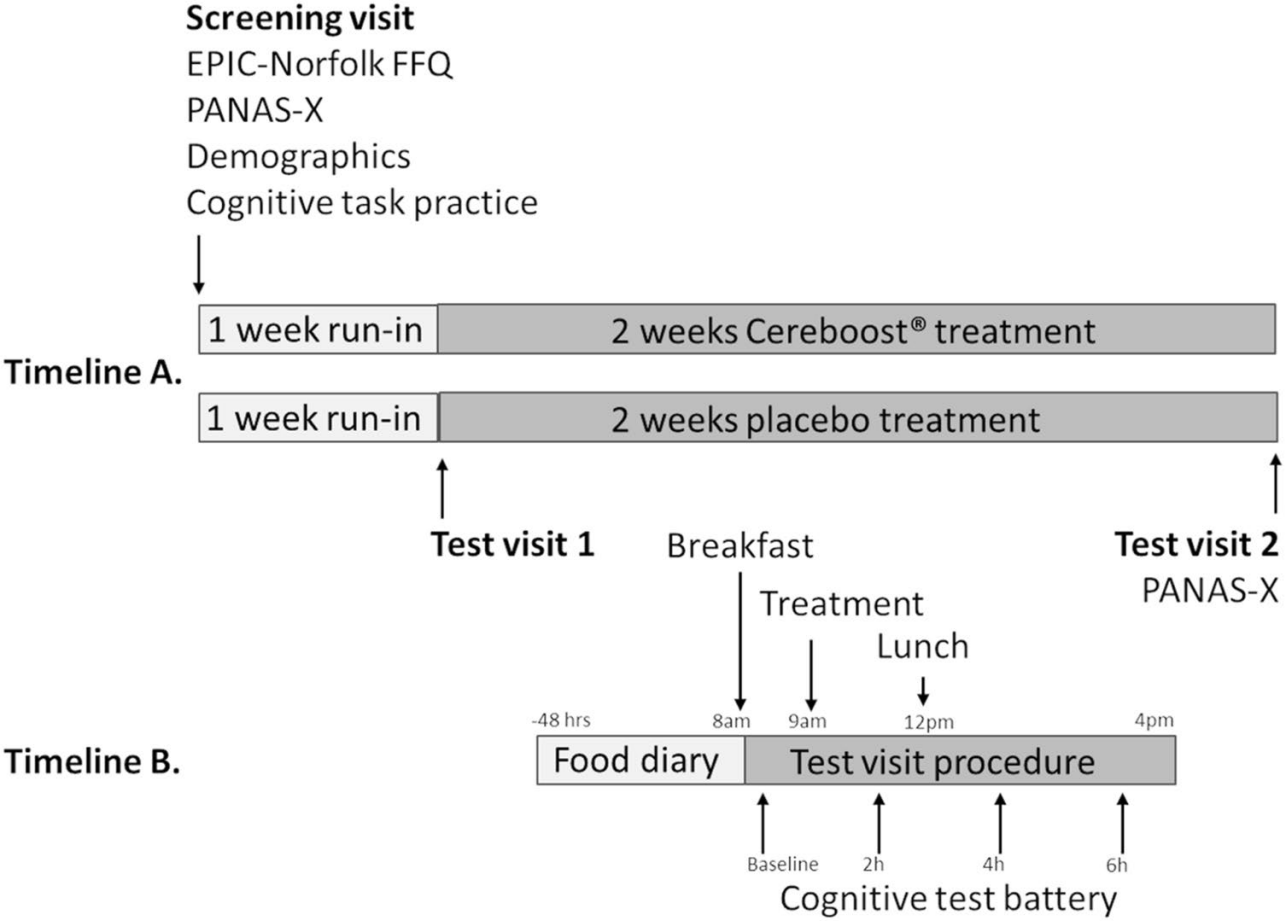
Study design: Timeline A represents the complete chronic study design; Timeline B represents the design of each acute or acute-on-chronic test visit (test visits 1 and 2, respectively). The cognitive test battery performed at each test visit consisted of PANAS-Now; immediate, and delayed word recall; Corsi blocks; attention network task; and switching task. In addition, PANAS-X was performed at the screening visit and again at baseline on test visit 2

The study design is shown in Fig. 1. Participants were randomized to receive either 200 mg Cereboost® treatment or a placebo, using a block design with a block size of 4 and an allocation ratio of 1:1. Participants and researchers were blind to the allocation which was implemented using sequentially numbered containers prepared independently by Naturex SA. Participants attended a screening visit where they were familiarised with the mood and cognitive tasks by completing the full task battery twice to reduce the likelihood of practice effects impacting the test data [29]. During a break between these two familiarisation sessions, participants completed demographic and habitual diet questionnaires. One week later, during the first test visit, mood and cognitive testing was performed at baseline (session 1), then 2 h, 4 h and 6 h following acute supplementation (sessions 2, 3, and 4, respectively). Participants then took supplements daily (1 capsule each morning with their breakfast) for a 2-week chronic intervention period, followed by a second test visit where mood and cognitive testing was repeated at baseline (session 5), then 2 h, 4 h, and 6 h following acute-on-chronic supplementation (sessions 6, 7, and 8, respectively). Participants attended each test visit in an overnight fasted state, having followed a low-polyphenol diet for 48 h.

### Treatments

The opaque Cereboost® capsules contained 200 mg of *P. quinquefolius* extract standardised for ginsenosides (10–12%) in accordance with patent US 8,968,800 B2. The placebo capsules were identical in appearance but contained only maltodextrin. Both sets of capsules were prepared by Naturex SA.

### Cognitive task battery

The computerized cognitive battery was presented using E-Prime 2.0 software (Psychology Software Tools, Pittsburgh, PA), and comprised tasks previously demonstrated to be sensitive to nutritional interventions. Cognitive domains targeted included attention, working memory, episodic memory, and mood. The battery took 30–40 min to complete at each test session. Ten equivalent versions of the battery, presented in counterbalanced order, were created to minimise practice effects between test sessions. The tasks are described in the order in which they appeared in the task battery.

#### PANAS-X (initially measured at the screening visit, then again at baseline on the final test visit)

The PANAS-X [30] is a self-report questionnaire consisting of 60 mood-related adjectives and is a recognised measure of trait mood. Participants rated their general mood over the last 2 weeks on a 5-point Likert Scale from “not at all” to “very much” for each item. Mood factor scores for Fear, Sadness, Guilt, Hostility, Shyness, Fatigue, Surprise, Joviality, Self-Assurance, Attentiveness, and Serenity were obtained, in addition to Positive Affect and Negative Affect scores like those derived for the PANAS-Now questionnaire.

#### Positive and Negative Affect Schedule Now (PANAS—Now)

The PANAS-Now questionnaire [31] is regarded as a reliable measure for examining current (or state) mood in non-clinical populations. During the task, participants were asked to rate 20 mood-related adjectives, indicating the extent to which they were currently experiencing that emotion on a 5-point Likert scale, ranging from “not at all” to “very much”. Half of the presented words related to positive emotions, the other half to negative emotions. Separate scores were obtained for positive affect and negative affect by summing ratings for all similarly valanced words. The PANAS-Now questionnaire was completed at the beginning (PANAS-Now 1) and end of the task battery (PANAS-Now 2), at each cognitive testing session.

#### Subjective mental fatigue

An additional item was added to the questionnaire to measure mental fatigue using a 9-point Likert scale [6]. Anchor points on the scale were ‘1. Not at all mentally fatigued’ and ‘9. Extremely mentally fatigued’. As with the PANAS-Now questionnaire, mental fatigue ratings were recorded at the beginning (mental fatigue 1) and end of the task battery (mental fatigue 2), at each cognitive testing session.

#### Immediate word recall

In this previously published episodic memory task [6], participants were visually presented with a sequential list of fifteen words. The participants were then given 1 min to type as many of the words as they could remember. A different word list, matched for linguistic familiarity, concreteness and frequency, was presented at each sitting of the task. The dependent variable was the number of correctly recalled words.

#### Corsi block tapping task

This visuo-spatial working memory task was a computerised version of the original Corsi Blocks task [32]. In the task, nine squares were presented on screen in a fixed position. Across multiple trials, a varying number of these squares flashed sequentially in quasi-random order. Participants viewed spatial sequences ranging from two to nine blocks. The participants were required to immediately repeat each sequence by clicking on the correct squares in the same order. The dependent variable was the number of correct sequences recalled.

#### Attention network task (ANT)

In this measure of executive function and attention [33], participants responded to the direction of a centrally presented target arrowhead by pressing the corresponding left and right arrow keys. Across multiple trials, the target stimulus was either flanked by arrows pointing in the same direction (congruent), or the opposite direction (incongruent). The number of flanking arrows also varied between trials (load). The task was completed in two blocks. During the second block, participants were distracted by noise through headphones. The dependent variables were reaction time and accuracy by congruency, load, and noise.

#### Rapid visual information processing task (RVIP)

In this sustained attention task [34], a series of digits were presented on screen in quick succession. The participant was required to monitor the digits for sequences of three consecutive even or three consecutive odd digits. Participants indicated the end of a target sequence by pressing the space bar as quickly as possible. The dependent variables were reaction time, correct responses, and commission errors. This task is reported to be an acetylcholine-sensitive task [6, 35] and has been shown to be sensitive to *P.ginseng* [36] and so was selected to be the primary outcome measure.

#### Switching task

This previously published task assessed executive function [37]. Participants viewed a circle with 8 equally spaced radii that formed 8 segments, 4 above and 4 below a bold line. A stimulus digit appeared sequentially in each segment in a clockwise direction. If the digit was located above the bold line, participants indicated whether the digit was odd or even using labelled arrow keys (left for odd, right for even). When the digit was below the bold line, participants again used the arrow keys to indicate whether the digit was higher or lower than 5 (left for higher, right for lower). Dependent variables were accuracy and reaction time.

#### Delayed word recall

As a measure of delayed episodic memory, participants were asked to type as many words as they could remember from the immediate recall word presentation. The task took place approximately 30 min after the initial presentation.

### Procedure

Testing took place in the Nutritional Psychology Unit at the School of Psychology & Clinical Languages, University of Reading. Each participant was required to visit the unit on three separate occasions (Fig. 1). The first visit was a screening visit where informed consent was obtained from all participants before completing a demographics questionnaire and a measure of habitual diet (EPIC-Norfolk food frequency questionnaire [38]). Participants were then given the opportunity to familiarise themselves with the cognitive tasks to minimise the impact of practice effects [29]. Participants returned approximately 1 week later for their first test visit and then again two weeks later for the final test visit. For 48 h prior to each test visit, participants were required to follow a low polyphenol diet and keep a food diary of everything they consumed as a record of dietary compliance. Alcohol and caffeine were restricted for 24 h only.

Participants arrived at the test visits in an overnight fasted state and were provided with a standardised breakfast (2 croissants and a glass of water) before performing baseline cognitive testing. Participants then received a capsule (either placebo or treatment depending on randomisation). Cognitive testing was then repeated 2 h, 4 h, and 6 h post intervention. A light standardized lunch was provided at the end of the 2 h session, which consisted of a cheese sandwich, a packet of ready salted crisps, and a glass of water. At the end of the first test visit, participants were provided with 2 weeks’ worth of placebo or treatment capsules to take home. They were instructed to take one every morning with breakfast and not to take one on the morning of the final test visit. Unused capsules were returned to monitor compliance. At the end of the final test visit, participants were awarded course credit for their participation.

### Safety

Participants were asked to report any negative health problems experienced throughout the course of the study. No issues were reported.

### Statistics

Data were analysed using SPSS statistics, version 25.0 (IBM Corporation). An intention-to-treat (ITT) analysis method incorporated all available data for each participant under the group they were assigned. Separate analyses were performed to test the acute, chronic, and acute-on-chronic effects of Cereboost® on all cognitive and mood-dependent variables. Further analysis compared acute effects with acute-on-chronic effects. Prior to each analysis, *z* scores were calculated to identify and remove any outlier data points with *z* score > 3.29. The procedure was performed twice for tasks with a small number of extreme outliers (ANT and Switching task) [52]. A linear marginal model (LMM), using an unstructured covariance matrix to model repeated response measures for each participant, was used to analyse all data. Baseline performance was included as a covariate.

*Acute:* Baseline data recorded at the first test visit (session 1) were entered as a covariate for the acute analysis. Treatment and placebo group scores were compared for data collected at sessions 2, 3, and 4, which corresponded to 2 h, 4 h, and 6 h post-prandial time points, respectively. Fixed factors included in the model were Baseline, Session, Treatment, and Session x Treatment interaction.

*Chronic:* For the chronic analysis, baseline data recorded at the first test visit (session 1) were entered as a covariate. Treatment and placebo group scores were compared at session 5. Fixed factors were Baseline and Treatment.

*Acute-on-chronic:* Baseline data recorded at the second test visit (session 5) were included as a covariate for the acute-on-chronic analysis. This analysis compared treatment and placebo group performance at sessions 6, 7, and 8 (again corresponding to 2 h, 4 h, and 6 h post-prandial timepoints). Fixed factors in the model were Baseline, Session, Treatment, and Session × Treatment interaction.

*Comparison between acute and acute-on-chronic:* For the additional analysis comparing acute data with acute-on-chronic data, a repeated baseline covariate was used incorporating baseline data from the first and second test visits (session 1 and session 5 data). Visit was included as an additional fixed factor in the model, alongside Baseline, Session, Treatment, and Session × Treatment interaction.

For the ANT task, congruency, load, noise and their respective treatment interactions were also included as fixed factors in all of the above analysis models. Post hoc, Bonferroni corrected, pairwise comparisons were used to investigate all significant treatment-related effects (*p* < 0.05). Cohen’s d effect sizes with 95% CI [LL, UL] were calculated for all significant pairwise comparisons between Cereboost® and placebo treatments.

## Results

**Fig. 2:**
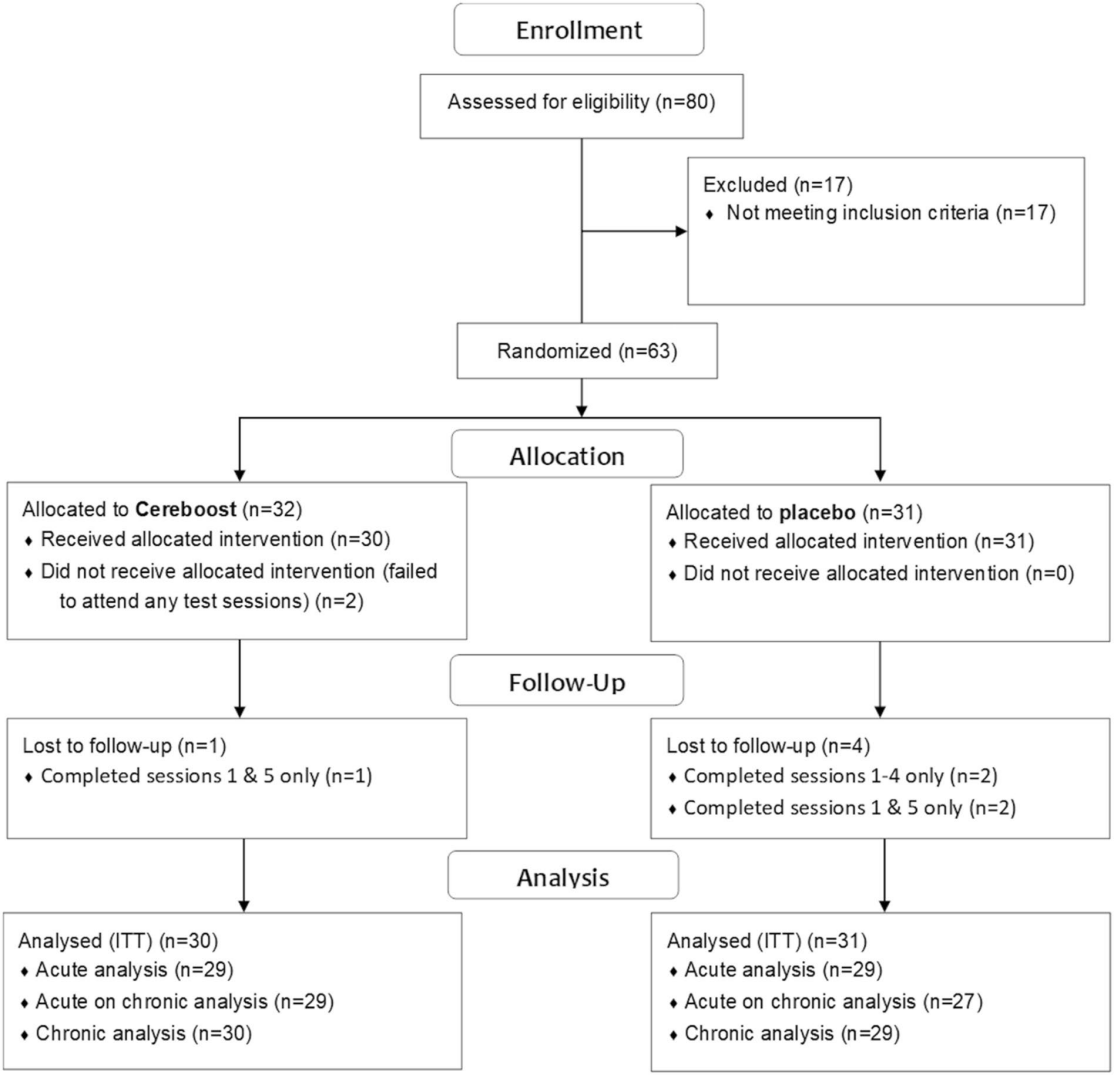
CONSORT diagram

Recruitment and data collection took place from May to August 2018. The trial ended when the required sample size was achieved. Sixty-three participants were recruited in total, however 2 participants failed to attend any test visits. The data for 61 participants were included in an ITT analysis. The CONSORT Diagram is shown in Fig. 2. Participant demographic information is shown in Table 1.

Only statistically significant findings from the primary and secondary outcome measures are presented here. Estimated marginal means and standard errors for all tasks and timepoints (including those with non-significant findings) are available in Supplemental Table S1.

### Acute analysis

**Fig. 3:**
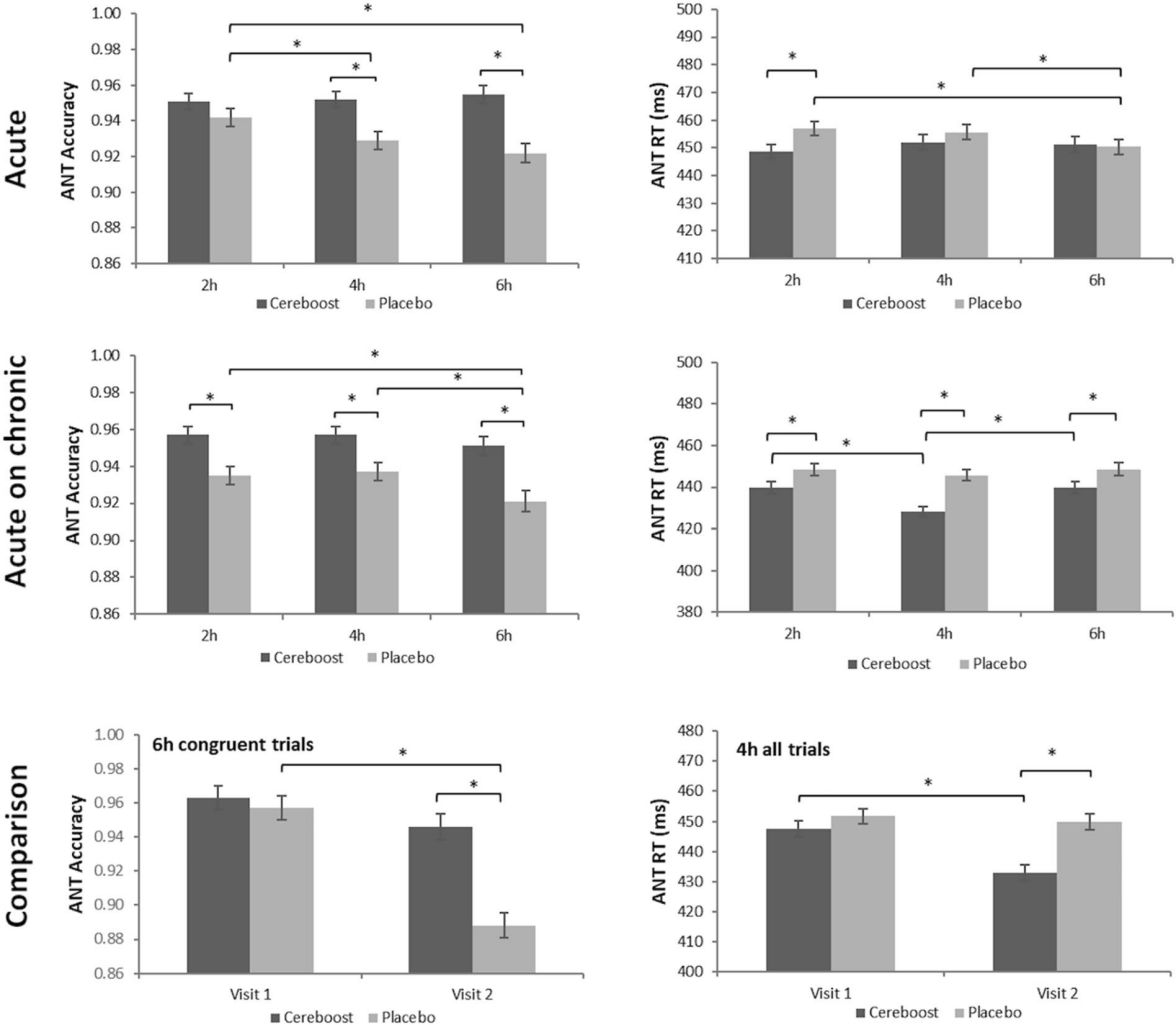
Maintenance of ANT accuracy and improved ANT reaction times observed in the immediate postprandial period following both acute and acute-on-chronic treatment with Cereboost® versus placebo. Comparison suggests that acute-on-chronic benefits observed at visit 2 are stronger than acute benefits observed at visit 1. Baseline values are included as a covariate, *indicates a significant difference between time points or treatments (*p* < 0.05), error bars represent mean standard error

Significant acute findings are shown in Fig. 3. For performance accuracy during the ANT task**,** the acute analysis revealed significant treatment [F(1,414.98) = 20.50, *p* < 0.001], and treatment × session [F(2,409.60) = 4.70, *p* = 0.010] effects. Pairwise comparisons revealed accuracy to be greater following Cereboost® treatment at the 4 h and 6 h test sessions (*p* = 0.001 and *p* < 0.001, respectively). Effect sizes were d = 0.96 [0.30, 1.39] at 4 h and *d* = 1.25 [0.68, 1.81] at 6 h. A significant reduction in accuracy was observed for the placebo treatment between the 2 h and 4 h sessions (*p* = 0.044), and between the 2 h and 6 h sessions (*p* = 0.001), suggestive of an acute maintenance effect of Cereboost® treatment. A significant treatment x congruency effect [F(1,414.96) = 20.63, *p* < 0.001] also revealed that Cereboost® treatment outperformed placebo treatment on incongruent trials (*p* < 0.001; *d* = 1.63 [1.03,2.22]), indicating a benefit on more cognitively challenging trials. For ANT reaction time, the acute analysis revealed a significant treatment × session interaction [F(2,433.97) = 3.57, *p* = 0.029]. Pairwise comparisons indicated faster reaction times for the Cereboost® treatment at the 2 h test session (*p* = 0.025; *d* = 0.60 [0.07,1.13]), compared with the placebo treatment. However, reaction times for the placebo treatment were observed to speed up between 2 and 6 h (*p* = 0.016), and between 4 and 6 h (*p* = 0.049). No acute benefits of Cereboost® were observed for the primary RVIP task, Corsi blocks, mood measures, the switching task, or immediate and delayed recall.

### Chronic analysis

**Fig. 4:**
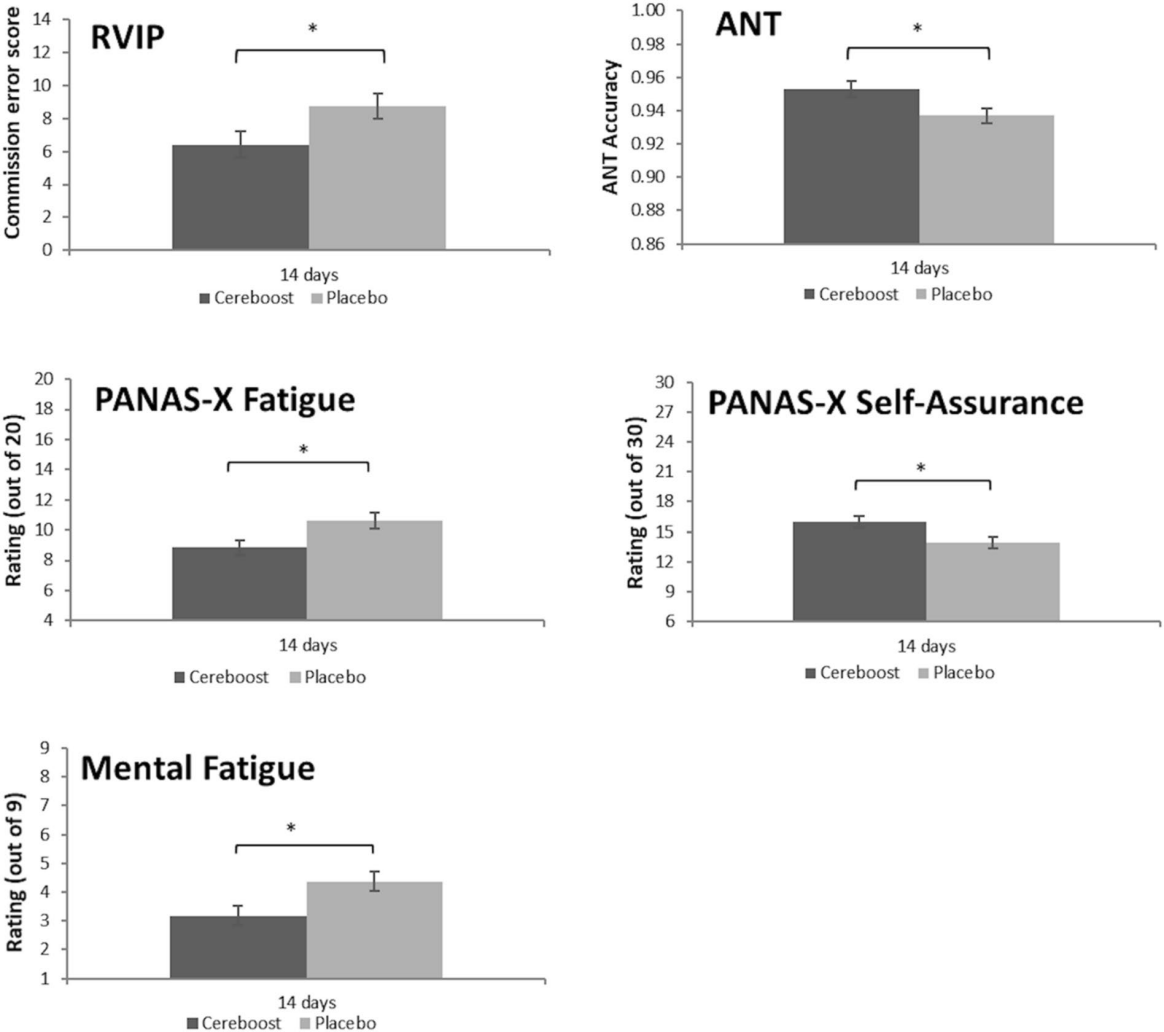
Group differences in RVIP, ANT, and subjective mood performance, observed at visit 2 following 14 days of chronic treatment with Cereboost® versus placebo. Baseline values are included as a covariate, *indicates a significant difference between time points or treatments (*p* < 0.05), error bars represent mean standard error

Significant chronic findings are shown in Fig. 4. For the primary outcome measure, chronic Cereboost® supplementation for 14 days resulted in significantly fewer RVIP commission errors compared to placebo [F(1,55) = 4.22, *p* = 0.045]; *d* = 0.55 [0.03,1.07]. Similarly, a significant effect of treatment on ANT accuracy was observed [F(1,433) = 6.29, *p* = 0.012] with Cereboost® outperforming placebo following 14 days treatment, *d* = 0.66 [0.14,1.18]. Unlike accuracy, however, no significant chronic effects were seen for ANT reaction time. The mood analysis revealed significant treatment effects for mental fatigue 1 [F(1,58) = 6.07, *p* = 0.017], which was mirrored in the PANAS-X fatigue measure [F(1,58) = 6.48, *p* = 0.014]. The PANAS-X self-assurance measure was also significant [F(1,58) = 5.84, *p* = 0.019]. In all cases, mood was better for the Cereboost® treatment compared with the placebo treatment. Effect sizes were d = 0.65 [0.13,1.18] for mental fatigue 1, d = 0.68 [0.15,1.20] for PANAS-X fatigue, and d = 0.64 [0.12,1.17] for PANAS-X self-assurance. No chronic effects were observed for Corsi blocks, the switching task, or immediate and delayed recall.

### Acute-on-chronic analysis

**Fig. 5:**
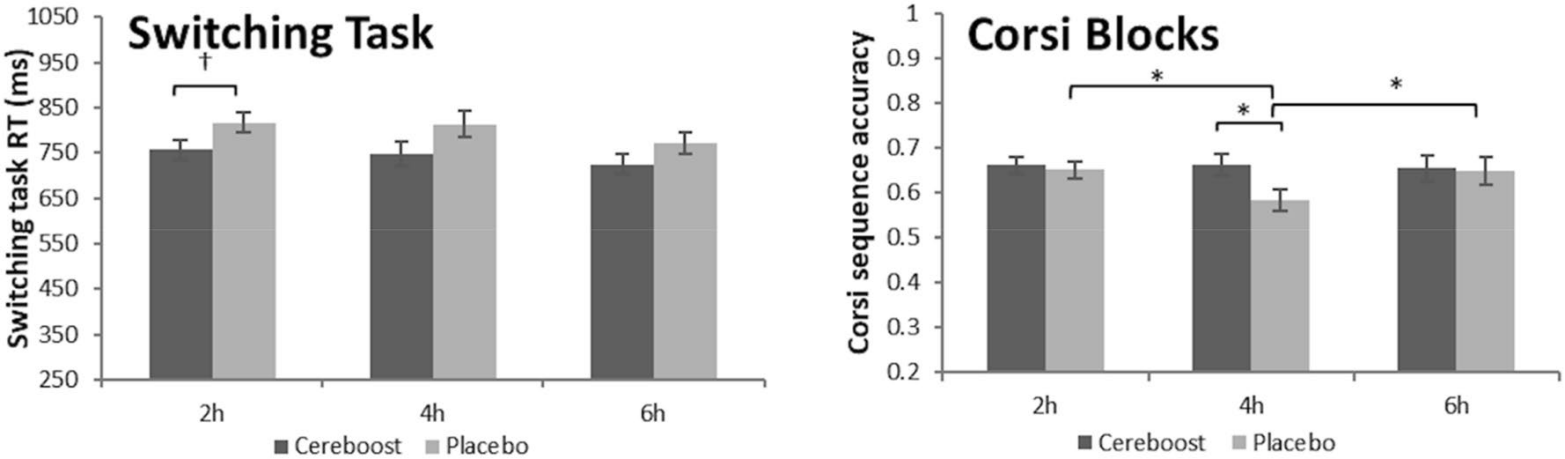
Maintenance of Corsi sequence accuracy and improved switching task reaction times observed at visit 2 in the immediate postprandial period following acute-on-chronic treatment with Cereboost® versus placebo. Baseline values are included as a covariate, *indicates a significant difference between time points or treatments (*p* < 0.05), error bars represent mean standard error

Significant acute-on-chronic findings are shown in Figs. 3 and 5. For the ANT task, a significant effect of treatment was observed for accuracy performance [F(1,369.09) = 20.76, *p* < 0.001]. Pairwise comparisons indicated significantly better performance for the Cereboost® treatment compared to placebo at all postprandial test sessions (2 h, *p* = 0.001; 4 h, *p* = 0.003; 6 h, p < 0.001). Effect sizes were d = 0.85 [0.30,1.39] at 2 h, d = 0.77 [0.23,1.31] at 4 h, and d = 1.05 [0.49–1.61] at 6 h. A significant reduction in accuracy was observed for the placebo treatment between the 2 h and 6 h sessions (*p* = 0.046), and between the 4 h and 6 h sessions (*p* = 0.035). For ANT reaction time, a significant treatment effect was observed [F(1,401.45) = 14.43, *p* < 0.001], with overall faster performance for the Cereboost® treatment compared with the placebo treatment. Pairwise comparisons revealed consistently faster performance for the Cereboost® treatment at all three test sessions (2 h, *p* = 0.032; 4 h, *p* < 0.001; 6 h, *p* = 0.036). Effect sizes were d = 0.59 [0.05,1.12] at 2 h, d = 1.30 [0.72,1.88] at 4 h, and d = 0.57 [0.04,1.11] at 6 h. Cereboost® reaction times sped up between 2 and 4 h (*p* < 0.001) but slowed again between 4 and 6 h (*p* = 0.001). The switching task revealed a significant effect of treatment for task reaction time [F(1,55.29) = 4.51, *p* = 0.038], with overall faster performance for the Cereboost® treatment compared with the placebo treatment. Pairwise comparisons indicated a trend for faster reaction times at the 2 h session (*p* = 0.064; d = 0.52 [-0.02,1.05]). The Corsi Blocks analysis revealed a significant treatment x session interaction for sequence accuracy [F(2,56) = 4.072, *p* = 0.022]. Pairwise comparisons indicated significantly better performance at 4 h for the Cereboost® treatment (*p* = 0.029; d = 0.61 [0.08,1.15]). Performance decreased between 2 and 4 h for the placebo treatment (*p* = 0.013), although it rose again between 4 and 6 h for the final test session of the study (*p* = 0.012), suggestive of more stable performance across the course of the day following Cereboost® treatment compared to placebo. No acute-on-chronic benefits of Cereboost® were observed for the primary RVIP task, mood measures, or immediate and delayed recall.

### Comparison between acute and acute-on-chronic benefits

Significant comparisons between acute and acute-on-chronic effects are shown in Fig. 3. While acute benefits were observed for only a single task (ANT), acute-on-chronic benefits were observed for 3 of the tasks (ANT, Corsi, Switching task) suggesting a possible enhancement of acute effects following a period of chronic supplementation. For the ANT task, accuracy scores as reported above were higher for Cereboost® treatment compared with placebo at two of the testing timepoints during acute testing at Visit 1, but these accuracy benefits were extended to all three time points during acute-on-chronic testing at Visit 2. Indeed, a direct comparison between acute and acute-on-chronic results for ANT accuracy revealed a significant treatment x session x congruency x visit interaction [F(11,800.28) = 2.86, *p* = 0.001], with a drop in performance between Visit 1 and Visit 2 for the placebo treatment at 6 h on congruent trials (*p* = 0.032); in contrast, a maintenance of performance was observed for the Cereboost® treatment. For ANT reaction times as reported above, responses were faster for Cereboost® treatment compared with placebo at only one of the testing timepoints during acute testing at Visit 1, but these reaction time benefits were extended to all three time points during acute-on-chronic testing at Visit 2. Further analysis of the comparison between acute and acute-on-chronic results for ANT reaction times revealed a significant treatment x session x visit interaction [F(2,817.96) = 23.05, *p* = 0.048], where faster performance was observed for the Cereboost® treatment at Visit 2 compared with Visit 1 (*p* < 0.001), specifically when looking at the 4 h test session.

### Experiment 2

## Methods

### Treatment and test chemicals

All chemicals were obtained from Sigma-Aldrich (Overijse, Belgium) unless stated otherwise. Naturex SA provided the Cereboost® treatment, which was tested at an in vitro dose of 200 mg per day, introduced as an aqueous solution.

### Simulator of the Human Intestinal Microbial Ecosystem (SHIME®)

The reactor configuration of the current experiment was adapted from the SHIME**®** protocol (ProDigest and Ghent University, Belgium) [39]. The present SHIME**®** setup consisted of a succession of three reactors simulating the different regions of the gastrointestinal tract, i.e., upper gastrointestinal tract including subsequent stomach and small intestinal simulation, proximal colon (PC) and distal colon (DC), respectively. Inoculum preparation, feeding regime, retention times, pH, temperature settings and nutritional medium composition have been previously outlined [40]. A faecal sample was obtained from a healthy, male donor, aged 34 years, and following an omnivorous Western diet. Upon introduction of the sample to the SHIME**®** system, a two-week stabilization period was initiated to allow the faecal microbiome to differentiate in the colonic reactors depending on the local environmental conditions. To simulate both the luminal and mucus-associated microbial community, mucin beads were included in the PC and DC to mimic the mucus layer as previously described [41]. Following the stabilization period, the experimental design included a two-week control period to determine baseline parameters. During these phases, the simulator was fed daily with a standard nutrient matrix. The control period was followed by a three-week treatment period where supplementation with 200 mg Cereboost® took place once per day in addition to the standard nutrient feed.

### Microbial metabolic activity

During the control and treatment period, samples for microbial metabolic activity were collected three times per week from the PC and DC. Analysis of SCFA levels, including acetate, propionate, butyrate, and total SCFA (including the previously mentioned SCFA as well as valerate, caproate, isobutyrate, isovalerate and isocaproate), was conducted as previously reported [42]*.*

### Microbial community analysis

Starting from the control period, samples for microbial community analysis were collected once per week from each colon vessel. DNA was isolated using a previously described method [43], with some minor modifications. Luminal DNA was extracted from pelleted bacterial cells obtained from a 1 mL sample, while mucosal DNA originated from 0.25 g mucin agar collected from the mucin beads. Homogenization was performed using a Fastprep-24 device (MP BioMedicals, Illkirch, France) performed twice for 40 s at 4 m/s with a resting period of 5 min between shakings.

Subsequently, quantitative polymerase chain reaction (qPCR) for the Firmicutes phylum, the Bacteroidetes phylum, *Akkermansia muciniphila*, *Bifidobacterium* spp. and *Lactobacillus* spp. was performed on a QuantStudio 5 Real-Time PCR system (Applied Biosystems, Foster City, CA USA). Each sample was analysed in technical triplicate and outliers (more than 1 C_T_ difference) were omitted. Different published qPCR methods were adopted for the Firmicutes and Bacteroidetes phyla [44], *Akkermansia muciniphila* [45], and *Lactobacillus* and *Bifidobacterium* spp [46, 47].

### Statistics

Statistical analysis was performed in GraphPad Prism 8.3.0. Normality of data and equality of the variances were confirmed with a Shapiro–Wilk test and a Brown–Forsythe test, respectively. For metabolic analysis parameters, normally distributed data with equal variances were analysed using ANOVA with a Tukey post hoc test. For microbial community composition, data were analysed using multiple independent t tests with correction for multiple comparisons using the Holm–Sidak method. Multiplicity adjusted p values were implemented. Differences were considered significant if *p* < 0.05.

## Results

### Analysis of the microbial metabolic activity

**Fig. 6:**
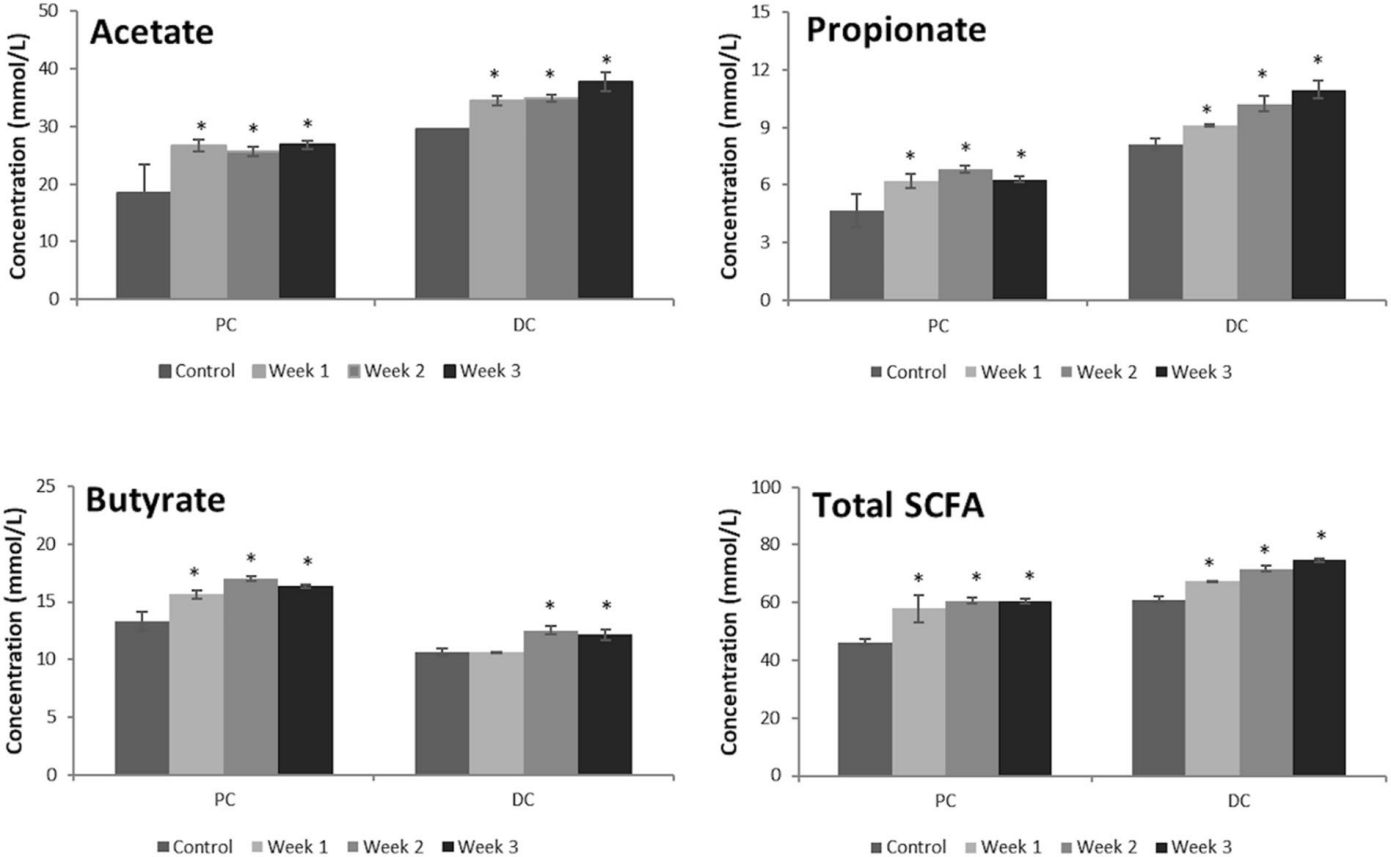
Increasing levels of acetate, propionate, butyrate, and total SCFA observed in a SHIME® simulation of the proximal and distal colon following a 3-week period of in vitro treatment with Cereboost®. *Indicates significant change in SCFA metabolic activity during weeks 1, 2, and 3 of treatment compared with the control period, error bars represent mean standard deviation

The SCFA profiles predominantly comprised acetate, propionate and butyrate. These are known to be the most abundant end points in colonic fermentation of dietary fibre [48] and provide conformation that the SHIME® simulation was working as expected. Changes in observed levels of these SCFAs across the control and treatment periods are shown in Fig. 6. ANOVA revealed significant time effects following dosing with Cereboost® for all SCFAs in both colon regions. Main effects of time are reported in Table 2. Post hoc tests revealed that Cereboost® significantly enhanced acetate levels compared to the control period in both colon regions (*p* = 0.043 and *p* = 0.001 at the end of the treatment period in the PC and DC, respectively). An average increase of 7.8 mmol/L (42.0%) and 6.2 mmol/L (21.2%) was observed in the PC and DC, respectively. In terms of propionate levels, it followed that the treatment with Cereboost® resulted in significantly increased propionate levels in the PC (*p* = 0.033 at the end of the treatment period), i.e., an average increase of 1.77 mmol/L (37.9%) as compared to the control period. In the DC, the Cereboost® treatment also resulted in significantly enhanced propionate levels (*p* = 0.001 at the end of the treatment period), with an average increase of 1.96 mmol/L (24.1%). Butyrate levels were significantly enhanced upon treatment with the test product in both colon regions (*p* = 0.002 and *p* = 0.019 at the end of the treatment period in the PC and DC, respectively), with strongest effects observed in the PC (i.e., an average increase of 3.0 mmol/L (22.6%)). Overall, the increased acetate, propionate and butyrate levels in the PC and DC resulted in significantly enhanced total SCFA levels in these colon regions (*p* = 0.005 and *p* = 0.001 at the end of the treatment period in the PC and DC, respectively).

**Table 2:** ANOVA outcomes for SCFA analysis

SCFA	Colon location	Time effect * F*(3,8)	*p* value
Acetate	Proximal	7.24	0.011
Acetate	Distal	38.77	< 0.001
Propionate	Proximal	11.34	0.003
Propionate	Distal	40.39	< 0.001
Butyrate	Proximal	22.82	< 0.001
Butyrate	Distal	16.26	0.001
Total SCFA	Proximal	19.00	0.001
Total SCFA	Distal	50.19	< 0.001

### Analysis of the microbial community composition

With respect to community composition, qPCR analysis was performed for targeted microbial groups (Table 3). *Lactobacillus* levels remained unaffected during treatment with Cereboost® in the luminal and mucosal PC. However, in the DC, Cereboost® supplementation resulted in enhanced *Lactobacillus* levels, with a clear trend in the mucosal environment (*p* = 0.066). *Bifidobacterium* levels decreased significantly in the mucosal environment of both the PC and DC during Cereboost® treatment (*p* = 0.004 and *p* < 0.001, respectively). Overall, Bacteroidetes levels remained largely unaffected during treatment with Cereboost®, with the exception a significant reduction in the mucosal DC (*p* = 0.001). Conversely, Firmicutes levels were significantly reduced in all colonic areas upon Cereboost® supplementation. Finally, while *Akkermansia muciniphila* remained below the limit of detection in the mucosal environment, treatment with Cereboost® resulted in a significant enrichment of *Akkermansia muciniphila* levels in the luminal DC (*p* = 0.002).

**Table 3:** Levels of microbial groups within the SHIME® simulation following treatment with Cereboost®

	Lumen				Mucus			
	Proximal colon		Distal colon		Proximal colon		Distal colon	
	Control	Cereboost®	Control	Cereboost®	Control	Cereboost®	Control	Cereboost®
*Lactobacillus*	5.7E + 07 ± 3.0E + 06	3.9E + 07 ± 2.5E + 07	6.3E + 06 ± 4.9E + 05	3.6E + 07 ± 2.4E + 07	2.4E + 08 ± 1.5E + 07	1.5E + 08 ± 9.1E + 07	2.9E + 06 ± 5.4E + 05	1.8E + 07 ± 9.1E + 06^†^
*Bifidobacterium*	1.5E + 10 ± 1.2E + 09	8.9E + 09 ± 4.2E + 09	6.0E + 09 ± 1.7E + 08	4.6E + 09 ± 1.9E + 09	1.2E + 10 ± 2.3E + 08	6.6E + 09 ± 1.9E + 09*	4.9E + 09 ± 1.1E + 08	2.0E + 09 ± 4.8E + 08*
*Bacteroidetes*	3.3E + 10 ± 1.3E + 09	2.3E + 10 ± 8.2E + 09	2.0E + 10 ± 1.5E + 08	1.5E + 10 ± 3.6E + 09	9.6E + 09 ± 1.4E + 08	6.2E + 09 ± 3.3E + 09	3.5E + 09 ± 6.6E + 07	1.7E + 09 ± 5.0E + 08*
*Firmicutes*	9.8E + 09 ± 3.8E + 08	5.0E + 09 ± 2.1E + 09*	8.6E + 09 ± 4.4E + 08	4.8E + 09 ± 1.5E + 09*	7.9E + 09 ± 3.6E + 08	3.2E + 09 ± 1.1E + 09*	5.4E + 09 ± 5.0E + 07	3.1E + 09 ± 4.5E + 08*
*Akkermansia muciniphila*	1.2E + 05 ± 8.9E + 03	1.0E + 05 ± 0.0E + 00	2.1E + 07 ± 2.4E + 06	3.2E + 07 ± 3.1E + 06*	Below LOQ	Below LOQ	Below LOQ	Below LOQ

Data presented as mean ± SD 16S rRNA gene copies/mL, * indicates significant difference between control and Cereboost® treatment (* p* < 0.05), ^†^ indicates non-significant trend (0.05 < * p* < 0.10)

## Discussion

The emerging pattern of cognitive results highlighted a maintenance of executive function and working memory performance in the immediate postprandial period following Cereboost® treatment, while chronic supplementation also offered some long-term benefits to performance accuracy and to subjective measures of mood and mental fatigue. In vitro Cereboost® treatment significantly increased SCFA (colonic acetate, propionate and butyrate levels), associated with increases in *Akkermansia muciniphila* and *Lactobacillus* levels.

The acute maintenance of performance on the ANT task is consistent with previous acute findings [6, 7]. In particular, these executive function effects are likely to result from activation of the prefrontal cortex (PFC), as observed in previous research [49]. Initially, acute effects were observed for ANT accuracy and reaction time at only one or two of the test sessions, but the acute-on-chronic effects observed for the ANT task were evident across the full day of cognitive testing, lasting up to 6 h. Additional acute-on-chronic effects were observed for the switching and Corsi tasks (measures of executive function and working memory, respectively), that were not apparent during the preliminary acute testing visit. This suggests that chronic supplementation may benefit the immediate response to the treatment. Chronic supplementation also offered some sustained long-term benefits. In particular, chronic supplementation was observed to decrease error rates on the RVIP task, though corresponding acute benefits were not observed for the same task. Similarly, no acute benefits of Cereboost® have been observed for RVIP performance in previous research [6]; therefore, chronic treatment seems to be necessary to observe benefits in this specific cognitive domain. Chronic supplementation with Cereboost® also offered some long-term benefits for subjective measures of mood (self-assurance) and mental fatigue. Such mood effects are likely to indirectly impact cognitive performance; increased self-assurance may reflect greater confidence to perform a cognitive task well, and lower levels of mental fatigue may help to maintain focus during a cognitive task. Indeed, similar mood and fatigue changes have been associated with better cognitive performance in previous research [50].

However, a notable observation from the current study was a lack of acute improvements to mood, and a lack of acute or chronic episodic memory benefits. Previously, following a 100 mg dose of *P. quinquefolius*, acute improvements in a Bond-Lader “Calmness” factor were observed in young adults [6]. However, the effect was not replicated in middle-aged adults when using a higher dose [7]. Therefore, it appears possible that *P. quinquefolius* may not facilitate broad improvements to mood at the dose used in the current study. Here, no changes to positive affect, negative affect or mental fatigue were observed during the immediate post-prandial period, although benefits to mental fatigue and self-assurance were subsequently observed after daily treatment for 14 days. Episodic memory effects have previously been observed following a similar acute dose [6], however differences in methodology (such as number of words presented, mode of presentation, and mode of recall) may explain differences in observed effects. A more sensitive episodic memory task such as Reys Auditory Verbal Learning Task (RAVLT) may be better suited to the investigation of acute memory changes, rather than the simplified task used here. Daily supplementation over a two-week period also failed to elicit an episodic memory effect in the current study. Short-term (working) memory effects were observed for the Corsi task during this time frame. However, benefits to long-term memory may require an extended supplementation period. It is also possible that the predominantly female sample tested may have influenced this memory outcome. Women are known to have an episodic memory advantage over men [51] and, although the memory recall scores obtained here don’t appear particularly high, memory improvements following Cereboost® treatment may, therefore, be more prevalent in males or in cognitively impaired populations such as older adults with age-related memory decline, rather than the cognitively intact young population tested here. The pre-clinical literature supports this theory, with a number of studies observing neuroprotective memory effects of ginseng in rodent models of ageing and neurodegenerative disease [4]. Therefore, it is recommended to investigate the memory effects of Cereboost® in an older adult population.

There are several possible underlying mechanisms of action that could explain the cognitive and mood changes that were observed in the current study following treatment with Cereboost®. For example, acetylcholine plays an important role in the modulation of cognition and affect, and acetylcholine-related pathways of neurotransmission are already known to be impacted by *P.quinquefolius* [13]. The chronic mood and fatigue effects observed here may therefore be due to facilitation of acetylcholine neurotransmission. Indeed, caffeine [52] and other natural extracts [53, 54] are known to promote benefits to mental fatigue, through inhibition of acetylcholine esterase [55]. With respect to cognition, RVIP was previously reported to be an acetylcholine-sensitive task following observed increases and decreases in task performance after treatment with an acetylcholine agonist (scopolamine) and an acetylcholine antagonist (nicotine), respectively [35]. In the current study, chronic treatment with Cereboost® seems to provide a beneficial effect on the RVIP task, thereby suggesting an acetylcholine-related mechanism of action for long-term cognitive improvements.

Acute benefits to RVIP performance were not observed in the current study. It is possible, however, that an acute 200 mg dose of Cereboost is insufficient to impact acetylcholine. Indeed, other previous research similarly failed to observe any effects of acute Cereboost® on RVIP performance [6]. One potential limitation of the current study design is that participants were required to abstain from caffeine for 24 h prior to test visits. The intention was to minimise the confounding effects of caffeine on cognitive function. However, habitual caffeine consumption was not determined and so participants may have been in caffeine withdrawal at the time of acute testing. This may have impacted any acute acetylcholine response, and so caffeine intake should be considered in any future research.

Emerging evidence also suggests that the gut microbiome may impact cognitive function and mood, in part due to the facilitation of effective digestion and metabolism of bioactive compounds in food. The health of the gut microbiome is largely dependent on diet quality. Diets high in fruit and vegetables are most beneficial for a healthy gut microbiota profile. The reported habitual fruit and vegetable consumption of the participants in the current study is in line with current UK recommended guidelines of 5 portions per day. However, these values were calculated via food frequency questionnaire and so may be overestimated [56]. Recent nutritional research also suggests that much higher fruit and vegetable intakes may be preferable for the optimisation of gut microbiota; a study investigating the benefits of increased fruit and vegetable intake observed benefits to gut health following the addition of up to 6 extra portions per day, on top of previous habitual intake levels (though these initial levels were not reported) [57]. Therefore, irrespective of the habitual intake of the student participants here, it remains possible that their microbiome was not optimal at the start of the in vivo study, with potential to improve following Cereboost® treatment. Indeed, during the present in vitro study using a donor sample from a young adult of similar demographic to the cognitive participants, significant increases in SCFA levels and *Akkermansia muciniphila* abundance, and a clear trend towards increased *Lactobacillus* levels, were observed across three weeks of Cereboost® supplementation. These improvements to the gut microbiome model provide a potential mechanism of action for improvements to cognitive function by facilitating not only the absorption and metabolism of bioactive compounds present in American ginseng, but potentially also the absorption of beneficial bioactive compounds found in habitually consumed foods.

It has been suggested that the gut microbiota play an important role in the bidirectional interactions between the central and the enteric nervous system, thereby likely affecting cognitive function [58]. Effective treatment paradigms for improving the gut microbiota profile include high-fibre diets, prebiotics, and probiotics [59]. In the current in vitro study, Cereboost® has demonstrated a prebiotic effect resulting in increased metabolite production as well as modulation of the microbial community composition. Therefore, it can be hypothesised that microbial modulation might be involved in the observed cognitive improvements following Cereboost® supplementation. Modulation of gut microbiota and resultant increases in SCFA production have been implicated in gut–brain signaling pathways including immune (neuroinflammatory response, mood), endocrine (learning and memory), vagal/neural (learning and memory) and humoral pathways (stress, neuroprotection) [60]. Indeed, this mechanism is consistent with both the chronic benefits of Cereboost® observed here, and the increase in acute benefits observed after a period of chronic supplementation.

As an important caveat, the supplementation period used for the SHIME® study was a week longer than the cognitive intervention in the current study. The changes to the gut microbial community were determined by comparing pre-treatment bacteria levels with post-treatment levels, but as sampling only occurred once per week during the treatment period, an average was calculated across the three weeks of treatment. This made it impossible to determine whether microbiota levels had significantly changed after two weeks. However, significant SCFA changes (generally associated with increased microbial activity) were observed after only one or two weeks, as shown in Fig. 6. Given these increased SCFA levels observed during this shorter period, it appears likely then that alterations to the gut microbiota remain a viable mechanism by which cognitive changes could be effected after only two weeks of daily Cereboost® treatment. One further caveat is that these changes to gut microbiome were observed in vitro*,* rather than in the cognitive study participants, and so it will be critical for future research to provide an in vivo link between changes in gut microbiota and cognitive changes in the same host (either animal or human). A further limitation in the current study is that participants were predominantly female, while the faecal donor was male, so future studies should seek to confirm the cognitive and gut microbiota effects of Cereboost® in a representative sample of both males and females. This would also allow determination of any sex differences in observed effects.

In conclusion, this study confirmed the promising effects of Cereboost® on cognitive function. Acute benefits to working memory and executive function were further improved following a two-week period of daily supplementation. Daily supplementation with Cereboost® also benefitted performance accuracy, mood and mental fatigue. Results from the concurrent in vitro study suggest a possible mechanism of action, via changes to the gut microbiome, potentially underlying the observed improvements to cognitive function. Chronic improvements to RVIP task performance also suggest that acetylcholine pathways of neurotransmission may be implicated. Further studies will be required to fully unravel the mechanisms involved.

## Supplementary Information

Below is the link to the electronic supplementary material.

Supplementary file1 (XLSX 16 kb)

## Data Availability

A supplementary data file is included.

## References

[1] Jia L, Zhao Y, Liang X-J (2009). Current evaluation of the millennium phytomedicine-ginseng (II): Collected chemical entities, modern pharmacology, and clinical applications emanated from traditional Chinese medicine. Curr Med Chem.

[2] Lieberman HR (2001). The effects of ginseng, ephedrine, and caffeine on cognitive performance, mood and energy. Nutr Rev.

[3] Kim J-S (2016). Investigation of phenolic, flavonoid, and vitamin contents in different parts of Korean Ginseng (Panax ginseng CA Meyer). Prevent Nutr Food Sci.

[4] Smith I, Williamson EM, Putnam S, Farrimond J, Whalley BJ (2014). Effects and mechanisms of ginseng and ginsenosides on cognition. Nutr Rev.

[5] Chen C-f, Chiou W-f, Zhang J-t (2008). Comparison of the pharmacological effects of Panax ginseng and Panax quinquefolium. Acta Pharmacol Sin.

[6] Scholey A, Ossoukhova A, Owen L, Ibarra A, Pipingas A, He K, Roller M, Stough C (2010). Effects of American ginseng (Panax quinquefolius) on neurocognitive function: an acute, randomised, double-blind, placebo-controlled, crossover study. Psychopharmacology.

[7] Ossoukhova A, Owen L, Savage K, Meyer M, Ibarra A, Roller M, Pipingas A, Wesnes K, Scholey A (2015). Improved working memory performance following administration of a single dose of American ginseng (*Panax quinquefolius* L.) to healthy middle-age adults. Human Psychopharmacol.

[8] Oshima Y, Sato K, Hikino H (1987). Isolation and hypoglycemic activity of quinquefolans A, B, and C, glycans of Panax quinquefolium roots. J Nat Prod.

[9] Vuksan V, Sievenpiper JL, Koo VY, Francis T, Beljan-Zdravkovic U, Xu Z, Vidgen E (2000). American ginseng (*Panax quinquefolius* L) reduces postprandial glycemia in nondiabetic subjects and subjects with type 2 diabetes mellitus. Arch Intern Med.

[10] Vuksan V, Sievenpiper JL, Wong J, Xu Z, Beljan-Zdravkovic U, Arnason JT, Assinewe V, Stavro MP, Jenkins AL, Leiter LA (2001). American ginseng (*Panax quinquefolius* L) attenuates postprandial glycemia in a time-dependent but not dose-dependent manner in healthy individuals. Am J Clin Nutr.

[11] Vuksan V, Stavro MP, Sievenpiper JL, Koo VY, Wong E, Beljan-Zdravkovic U, Francis T, Jenkins AL, Leiter LA, Josse RG (2000). American ginseng improves glycemia in individuals with normal glucose tolerance: effect of dose and time escalation. J Am Coll Nutr.

[12] Sloley BD, Pang P, Huang B-H, Ba F, Li FL, Benishin CG, Greenshaw AJ, Shan JJ (1999). American ginseng extract reduces scopolamine-induced amnesia in a spatial learning task. J Psychiatry Neurosci.

[13] Shin K, Guo H, Cha Y, Ban Y-H, Seo DW, Choi Y, Kim T-S, Lee S-P, Kim J-C, Choi E-K (2016). Cereboost^TM^, an American ginseng extract, improves cognitive function via up-regulation of choline acetyltransferase expression and neuroprotection. Regul Toxicol Pharmacol.

[14] Salim KN, McEwen BS, Chao HM (1997). Ginsenoside Rb1 regulates ChAT, NGF and trkA mRNA expression in the rat brain. Mol Brain Res.

[15] Benishin CG, Lee R, Wang LCH, Liu HJ (1991). Effects of ginsenoside Rb1 on central cholinergic metabolism. Pharmacology.

[16] Hasselmo ME, Sarter M (2011). Modes and models of forebrain cholinergic neuromodulation of cognition. Neuropsychopharmacology.

[17] Ferreira-Vieira H, T, M Guimaraes I, R Silva F, M Ribeiro F, (2016). Alzheimer's disease: targeting the cholinergic system. Curr Neuropharmacol.

[18] Sünram-Lea S, Birchall R, Wesnes K, Petrini O (2005). The effect of acute administration of 400 mg of Panax ginseng on cognitive performance and mood in healthy young volunteers. Curr Top Nutraceut Res.

[19] Wang C-Z, Kim KE, Du G-J, Qi L-W, Wen X-D, Li P, Bauer BA, Bissonnette MB, Musch MW, Chang EB (2011). Ultra-performance liquid chromatography and time-of-flight mass spectrometry analysis of ginsenoside metabolites in human plasma. Am J Chin Med.

[20] Oh J, Kim J-S (2016). Compound K derived from ginseng: neuroprotection and cognitive improvement. Food Funct.

[21] Kim H-K (2013). Pharmacokinetics of ginsenoside Rb1 and its metabolite compound K after oral administration of Korean Red Ginseng extract. J Ginseng Res.

[22] Hasegawa H (2004). Proof of the mysterious efficacy of ginseng: basic and clinical trials: metabolic activation of ginsenoside: deglycosylation by intestinal bacteria and esterification with fatty acid. J Pharmacol Sci.

[23] Wan JY, Wang CZ, Zhang QH, Liu Z, Musch MW, Bissonnette M, Chang EB, Li P, Qi LW, Yuan CS (2017). Significant difference in active metabolite levels of ginseng in humans consuming Asian or Western diet: the link with enteric microbiota. Biomedical Chromatography.

[24] Wang C-Z, Yu C, Wen X-D, Chen L, Zhang C-F, Calway T, Qiu Y, Wang Y, Zhang Z, Anderson S (2016). American ginseng attenuates colitis-associated colon carcinogenesis in mice: Impact on gut microbiota and metabolomics. Cancer Prev Res.

[25] Song M-Y, Kim B-S, Kim H (2014). Influence of Panax ginseng on obesity and gut microbiota in obese middle-aged Korean women. J Ginseng Res.

[26] Davidson GL, Cooke AC, Johnson CN, Quinn JL (2018). The gut microbiome as a driver of individual variation in cognition and functional behaviour. Philos Trans R Soc B.

[27] Leeming ER, Johnson AJ, Spector TD, Le Roy CI (2019). Effect of diet on the gut microbiota: rethinking intervention duration. Nutrients.

[28] Neale C, Camfield D, Reay J, Stough C, Scholey A (2013). Cognitive effects of two nutraceuticals G inseng and B acopa benchmarked against modafinil: a review and comparison of effect sizes. Br J Clin Pharmacol.

[29] Bell L, Lamport DJ, Field DT, Butler LT, Williams CM (2018). Practice effects in nutrition intervention studies with repeated cognitive testing. Nutr Healthy Aging.

[30] Watson D, Clark LA (1999) The PANAS-X: manual for the positive and negative affect schedule-expanded form.

[31] Watson D, Clark LA, Tellegen A (1988). Development and validation of brief measures of positive and negative affect: the PANAS scales. J Pers Soc Psychol.

[32] Whyte AR, Cheng N, Fromentin E, Williams CM (2018). A randomized, double-blinded, placebo-controlled study to compare the safety and efficacy of low dose enhanced wild blueberry powder and wild blueberry extract (ThinkBlue^TM^) in maintenance of episodic and working memory in older adults. Nutrients.

[33] Whyte AR, Schafer G, Williams CM (2017). The effect of cognitive demand on performance of an executive function task following wild blueberry supplementation in 7 to 10 years old children. Food Funct.

[34] Watson AW, Haskell-Ramsay CF, Kennedy DO, Cooney JM, Trower T, Scheepens A (2015). Acute supplementation with blackcurrant extracts modulates cognitive functioning and inhibits monoamine oxidase-B in healthy young adults. Journal of functional foods.

[35] Wesnes K, Warburton DM (1984). Effects of scopolamine and nicotine on human rapid information processing performance. Psychopharmacology.

[36] Reay JL, Kennedy DO, Scholey AB (2006). Effects of Panax ginseng, consumed with and without glucose, on blood glucose levels and cognitive performance during sustained ‘mentally demanding’tasks. J Psychopharmacol.

[37] Miller MG, Hamilton DA, Joseph JA, Shukitt-Hale B (2018). Dietary blueberry improves cognition among older adults in a randomized, double-blind, placebo-controlled trial. Eur J Nutr.

[38] Mulligan AA, Luben RN, Bhaniani A, Parry-Smith DJ, O'Connor L, Khawaja AP, Forouhi NG, Khaw K-T (2014) A new tool for converting food frequency questionnaire data into nutrient and food group values: FETA research methods and availability. BMJ open 4 (3): 004503 10.1136/bmjopen-2013-004503 PMC3975761 24674997

[39] Molly K, Woestyne MV, Verstraete W (1993). Development of a 5-step multi-chamber reactor as a simulation of the human intestinal microbial ecosystem. Appl Microbiol Biotechnol.

[40] Possemiers S, Verthé K, Uyttendaele S, Verstraete W (2004). PCR-DGGE-based quantification of stability of the microbial community in a simulator of the human intestinal microbial ecosystem. FEMS Microbiol Ecol.

[41] Van den Abbeele P, Roos S, Eeckhaut V, MacKenzie DA, Derde M, Verstraete W, Marzorati M, Possemiers S, Vanhoecke B, Van Immerseel F (2012). Incorporating a mucosal environment in a dynamic gut model results in a more representative colonization by lactobacilli. Microb Biotechnol.

[42] De Weirdt R, Possemiers S, Vermeulen G, Moerdijk-Poortvliet TC, Boschker HT, Verstraete W, Van de Wiele T (2010). Human faecal microbiota display variable patterns of glycerol metabolism. FEMS Microbiol Ecol.

[43] Boon N, Top EM, Verstraete W, Siciliano SD (2003). Bioaugmentation as a tool to protect the structure and function of an activated-sludge microbial community against a 3-chloroaniline shock load. Appl Environ Microbiol.

[44] Guo X, Xia X, Tang R, Zhou J, Zhao H, Wang K (2008). Development of a real-time PCR method for Firmicutes and Bacteroidetes in faeces and its application to quantify intestinal population of obese and lean pigs. Lett Appl Microbiol.

[45] Collado MC, Derrien M, Isolauri E, de Vos WM, Salminen S (2007). Intestinal integrity and Akkermansia muciniphila, a mucin-degrading member of the intestinal microbiota present in infants, adults, and the elderly. Appl Environ Microbiol.

[46] Furet J-P, Firmesse O, Gourmelon M, Bridonneau C, Tap J, Mondot S, Doré J, Corthier G (2009). Comparative assessment of human and farm animal faecal microbiota using real-time quantitative PCR. FEMS Microbiol Ecol.

[47] Rinttilä T, Kassinen A, Malinen E, Krogius L, Palva A (2004). Development of an extensive set of 16S rDNA-targeted primers for quantification of pathogenic and indigenous bacteria in faecal samples by real-time PCR. J Appl Microbiol.

[48] Cummings JH (1981). Short chain fatty acids in the human colon. Gut.

[49] White DJ, Camfield DA, Ossoukhova A, Savage K, Le Cozannet R, Fança-Berthon P, Scholey A (2020). Effects of Panax quinquefolius (American ginseng) on the steady state visually evoked potential during cognitive performance. Hum Psychopharmacol.

[50] Scholey AB, French SJ, Morris PJ, Kennedy DO, Milne AL, Haskell CF (2010). Consumption of cocoa flavanols results in acute improvements in mood and cognitive performance during sustained mental effort. J Psychopharmacol.

[51] Asperholm M, Högman N, Rafi J, Herlitz A (2019). What did you do yesterday? A meta-analysis of sex differences in episodic memory. Psychol Bull.

[52] Cappelletti S, Daria P, Sani G, Aromatario M (2015). Caffeine: cognitive and physical performance enhancer or psychoactive drug?. Curr Neuropharmacol.

[53] Spasov A, Wikman G, Mandrikov V, Mironova I, Neumoin V (2000). A double-blind, placebo-controlled pilot study of the stimulating and adaptogenic effect of Rhodiola rosea SHR-5 extract on the fatigue of students caused by stress during an examination period with a repeated low-dose regimen. Phytomedicine.

[54] Darbinyan V, Kteyan A, Panossian A, Gabrielian E, Wikman G, Wagner H (2000). Rhodiola rosea in stress induced fatigue—a double blind cross-over study of a standardized extract SHR-5 with a repeated low-dose regimen on the mental performance of healthy physicians during night duty. Phytomedicine.

[55] Hillhouse B, Ming DS, French C, Towers G (2004). Acetylcholine esterase inhibitors in Rhodiola rosea. Pharm Biol.

[56] Bingham S, Luben R, Welch A, Low YL, Khaw KT, Wareham N, Day N (2008). Associations between dietary methods and biomarkers, and between fruits and vegetables and risk of ischaemic heart disease, in the EPIC Norfolk Cohort Study. Int J Epidemiol.

[57] Klinder A, Shen Q, Heppel S, Lovegrove JA, Rowland I, Tuohy KM (2016). Impact of increasing fruit and vegetables and flavonoid intake on the human gut microbiota. Food Funct.

[58] Carabotti M, Scirocco A, Maselli MA, Severi C (2015). The gut-brain axis: interactions between enteric microbiota, central and enteric nervous systems. Annals of gastroenterology: quarterly publication of the Hellenic Society of Gastroenterology.

[59] Sun Y, Baptista LC, Roberts LM, Jumbo-Lucioni P, McMahon LL, Buford TW, Carter CS (2020). The gut microbiome as a therapeutic target for cognitive impairment. The Journals of Gerontology: Series A.

[60] Dalile B, Van Oudenhove L, Vervliet B, Verbeke K (2019) The role of short-chain fatty acids in microbiota–gut–brain communication. Nature Reviews Gastroenterology & Hepatology:1 10.1038/s41575-019-0157-3 31123355

